# Overcoming Barriers to Yoga Implementation in Cancer Care: A Call for Integration

**DOI:** 10.7759/cureus.69292

**Published:** 2024-09-12

**Authors:** Selvaraj Giridharan

**Affiliations:** 1 Oncology, Tawam Hospital, Al Ain, ARE

**Keywords:** barriers, cancer care, complementary cancer care, complementary therapy, qol, quality of life (qol), research, yoga, yoga therapy, barriers

## Abstract

Integrating yoga into cancer care is gaining attention due to its potential benefits, including improved quality of life, reduced treatment-related side effects, and enhanced psychological health. However, its adoption in clinical practice is hindered by various barriers. These barriers include limited access and availability, particularly in rural or low-resource settings; cultural and psychological misconceptions; skepticism among healthcare providers; and a lack of standardized protocols. Additionally, the perceived lack of rigorous scientific evidence and insufficient funding for research pose significant challenges to its acceptance in oncology care. This editorial explores these barriers and proposes solutions to promote the integration of yoga into standard cancer treatment. By addressing these challenges, yoga can move from being an adjunctive therapy to becoming a core component of comprehensive cancer care, ultimately improving patient outcomes and quality of life.

## Editorial

Introduction

In recent years, integrative oncology has gained substantial attention as a comprehensive approach to cancer care. This holistic method focuses not only on disease treatment but also on enhancing overall patient well-being. Among the various complementary therapies, yoga has emerged as a promising practice offering numerous benefits for cancer patients. The advantages of yoga include improved quality of life, reduced treatment-related side effects, enhanced psychological health, and its cost-effectiveness and ease of practice, making it accessible to a broad range of patients [1]. Despite accumulating evidence supporting yoga's efficacy in oncology, its integration into clinical practice remains constrained. This editorial seeks to delineate the barriers impeding the widespread adoption of yoga in cancer care and proposes strategies to overcome these challenges. By addressing these obstacles, we can pave the way for a more integrative approach to cancer treatment, wherein yoga transcends being merely an adjunctive therapy to become a cornerstone of comprehensive patient care.

Understanding the challenges

Access and Availability

Access to yoga therapy in cancer care is limited, particularly for patients in rural or low-resource settings. Urban areas may offer a variety of yoga classes and specialized instructors, but many cancer patients live in regions where such services are scarce or absent. This lack of access exacerbates health disparities, depriving a substantial portion of the cancer patient population of the potential therapeutic benefits of yoga. Moreover, the availability of trained yoga instructors who specialize in working with cancer patients is limited, and the lack of institutional support within healthcare settings is a significant obstacle. Many hospitals and oncology centers do not provide on-site yoga therapy, requiring patients to seek these services independently. A cross-sectional survey by Gonzalez et al. revealed that many cancer patients prefer accessible and adaptable yoga practices, such as online or group sessions [2].

Cultural and Psychological Barriers

Cultural and psychological barriers significantly influence the participation of some cancer patients in yoga. These barriers stem from misconceptions about yoga, concerns regarding its suitability during cancer treatment, and diverse cultural perspectives. For instance, a study of African American cancer survivors showed that fears of stigma and concerns about culturally inappropriate instruction deterred participation. Understanding and addressing these barriers is crucial to broadening the appeal of yoga as an inclusive option for all cancer patients. One prevalent cultural barrier is the association of yoga with specific religious or spiritual practices. In certain communities, yoga is perceived as incompatible with particular religious beliefs, deterring patient engagement. Furthermore, media portrayals of yoga as a physically demanding activity for young, fit individuals create the misconception that it is unsuitable for individuals with physical limitations or older adults, a concern for many cancer patients.

Psychological barriers are also significant. The fatigue, pain, and emotional challenges experienced by cancer patients during treatment can make the idea of engaging in physical activity, including yoga, overwhelming. There may also be a fear of exacerbating symptoms or causing harm, particularly when patients lack an understanding of how yoga can be adapted to their needs during treatment. Another psychological barrier is the stigma associated with seeking complementary therapies. Some patients fear judgment from healthcare providers or peers for choosing yoga, worrying that it may be perceived as a substitute for conventional medical treatment rather than a complementary practice. This fear can hinder patients from considering yoga as an option.

Integration Into Conventional Care

Integrating yoga into standard cancer care poses significant challenges due to institutional, professional, and systemic obstacles. The primary barriers include skepticism among healthcare providers, lack of standardized protocols, and limited institutional support. Despite a growing body of evidence supporting the benefits of yoga for cancer patients, many healthcare providers remain hesitant to recommend or integrate yoga into treatment plans. This skepticism often stems from a perception that yoga lacks sufficient scientific validation, particularly in comparison to more established medical interventions. Additionally, some providers may not be familiar with recent research or may prioritize conventional treatments they believe have more direct or immediate effects on cancer outcomes.

The absence of standardized protocols for the use of yoga in oncology also contributes to its limited adoption. Unlike medications or surgical interventions, yoga does not have clearly defined guidelines specifying which practices are most appropriate for different types of cancer, stages of disease, or treatment phases. This lack of standardization creates ambiguity and inconsistency in how yoga is recommended and practiced within healthcare settings, making it difficult for providers to incorporate it confidently into care plans.

Furthermore, institutional support for on-site yoga programs is often restricted by budgetary constraints, space limitations, and a shortage of trained personnel who specialize in yoga for cancer care [3]. Many healthcare facilities may not have the financial resources to hire certified yoga therapists or allocate space for yoga sessions, viewing it instead as an ancillary service that patients must seek independently. This perception of yoga as an optional, out-of-pocket service rather than an integrated component of cancer care limits its accessibility, particularly for patients who might benefit most but cannot afford additional expenses.

Research and Evidence

The utilization of yoga in cancer care is frequently hindered by the perceived insufficiency of robust scientific evidence validating its efficacy. While a mounting body of research suggests that yoga can improve quality of life, mitigate treatment-related side effects, and enhance psychological well-being for cancer patients, numerous healthcare providers remain circumspect, emphasizing the necessity for more rigorous, high-quality studies to substantiate its effectiveness [4]. Several challenges contribute to this prevailing perception. Many studies examining yoga's benefits in cancer care have methodological constraints, including small sample sizes, absence of randomization, and heterogeneous patient cohorts, complicating the generalization of findings [5]. Moreover, the diverse array of outcomes measured in these studies, ranging from physical symptoms like pain and fatigue to psychological factors such as anxiety and depression, engenders inconsistencies in the evidence, rendering it arduous for healthcare providers to ascertain yoga's efficacy conclusively.

The paucity of funding for research on complementary therapies like yoga also poses a substantial obstacle. Conventional funding sources often precede pharmaceutical or traditional medical interventions, resulting in the underfunding of yoga studies. This financial constraint curtails the magnitude and sphere of research, yielding a dearth of large-scale, high-quality studies that could compellingly substantiate the benefits of yoga in cancer care. Another challenge is the acceptance of qualitative outcomes, such as patient-reported quality of life, within the broader medical community. While these outcomes are indispensable for comprehending yoga's holistic impact, they are frequently undervalued compared to more quantitative measures, such as tumor response rates or survival outcomes, perpetuating skepticism regarding yoga's pertinence in oncology care.

Pathways to integration

To effectively integrate yoga into cancer care, several strategies should be pursued to address access, availability, and cultural barriers.

Enhancing Access and Availability

Healthcare providers should prioritize expanding telehealth yoga programs, which have proven effective during the COVID-19 pandemic by offering flexible, remote access to therapy. Compared with other complementary therapies, yoga is particularly advantageous due to its low cost, minimal equipment requirements, and adaptability to individual needs, making it accessible to a broader range of patients. Community-based programs offering subsidized or free yoga classes for cancer patients can further bridge the access gap, ensuring that financial limitations do not prevent patients from benefiting from this practice. Integrating yoga into standard oncology care through on-site programs enhances accessibility and provides a safe environment under the supervision of healthcare professionals.

Overcoming Cultural and Psychological Barriers

Overcoming cultural and psychological barriers requires active involvement from healthcare providers. Unlike some complementary therapies that may be perceived as more invasive or requiring specialized facilities, yoga is noninvasive, adaptable, and can be practiced in various settings. Culturally sensitive education that reframes yoga as a therapeutic practice suitable for all, regardless of age, fitness level, or religious belief, is crucial. Healthcare providers should proactively discuss yoga as part of a comprehensive cancer care plan, emphasizing gentle, adaptable practices tailored to cancer patients. Their role in promoting open communication and dispelling myths can help reduce fears and stigma, encouraging broader adoption.

Developing Standardized and Tailored Yoga Modules

To facilitate integration into conventional care, healthcare institutions should foster collaborative efforts among healthcare professionals. This includes developing standardized, evidence-based guidelines for yoga therapy in oncology. Additionally, creating validated and tailored yoga modules for specific cancer types, such as breast cancer, lung cancer, colorectal cancer, and prostate cancer, can enhance the effectiveness and acceptance of yoga therapy. These modules should be developed with input from yoga therapists, oncologists, and other healthcare professionals to ensure they are practical and applicable to the unique needs of different cancer types and stages.

Strengthening the Research Base

Strengthening the research base is essential for the broader adoption of yoga in oncology. Future studies should prioritize large-scale, multicentric, randomized controlled trials (RCTs) with standardized protocols to enhance the reliability of findings and include long-term assessments of yoga’s effects on cancer survivors. Such multicentric studies can provide diverse patient data, making results more generalizable across different populations. Emphasizing patient-reported outcomes, such as quality of life, symptom management, and overall well-being, will provide a more comprehensive understanding of yoga’s benefits. Additionally, increased funding and support for yoga research will help build a more substantial evidence base, encouraging wider acceptance and integration.

Highlighting Unique Benefits of Yoga

Yoga offers several unique benefits compared to other complementary therapies, including its ability to address both physical and psychological aspects of cancer care. It has been shown to reduce anxiety, improve mood, enhance sleep quality, and alleviate physical symptoms such as pain and fatigue. Unlike some other therapies, yoga can be easily tailored to accommodate different physical abilities, making it suitable for a wide range of patients. Its focus on breathwork, mindfulness, and relaxation also aligns well with the needs of cancer patients who may be dealing with stress, anxiety, and emotional challenges.

Conclusion

Overcoming the barriers to yoga implementation in cancer care is possible and necessary. By addressing access issues, cultural and psychological misconceptions, integration challenges, and research gaps, we can make yoga a standard component of comprehensive cancer care. As interest in integrative oncology grows, embracing yoga will ensure that all patients experience its profound benefits throughout their cancer journey.

## References

[1] Niu N, Huang R, Zhao J, Zeng Y (2024). Health benefits of yoga for cancer survivors: an updated systematic review and meta-analysis. Asia Pac J Oncol Nurs.

[2] Gonzalez M, Grant S, de Manincor M, Lacey J, Sarris J (2023). Cancer survivors' experiences, barriers and preferences with yoga: a cross-sectional survey to inform a yoga intervention. Explore (NY).

[3] Larbi OM, Jiang C, McLane B (2021). Interest and willingness to pay for Integrative therapies of patients with cancer and caregivers. JCO Oncol Pract.

[4] Slocum-Gori S, Howard AF, Balneaves LG, Kazanjian A (2013). Investigating the perceived feasibility of integrative medicine in a conventional oncology setting: yoga therapy as a treatment for breast cancer survivors. Integr Cancer Ther.

[5] Desai K, Bao T, Li QS (2021). Understanding interest, barriers, and preferences related to yoga practice among cancer survivors. Support Care Cancer.

